# Study of silver aerosol source term at different specific internal energy input from HE detonation devices

**DOI:** 10.1038/s41598-022-07180-w

**Published:** 2022-02-24

**Authors:** Song Kefeng, Shi Yaqin, Liu Kun, Su Luochuan, Li Bo, Liu Wei, Wang Penglai, Yi Chenhong, Zhang Yajun, Ma Qingpeng, Hu Haibo, Liu Wenjie

**Affiliations:** 1 China Academy of Engineering Physics Institute of Fluid Physics, Mianyang, Sichuan China; 2 China Academy of Engineering Physics, Mianyang, Sichuan China

**Keywords:** Applied physics, Particle physics

## Abstract

Radioactive aerosols harmful to humans are often produced in nuclear accidents, and their source term characteristics (total volume and particle size distribution) and dispersion patterns have important implications for accident response and hazard assessment. However, experimental studies of radioactive aerosols cannot be directly conducted in open space due to the hazardous nature of radioactive aerosols. In this study, silver was used instead of plutonium to study the aerosol source term under different specific internal energy inputs (SIEI) to simulate a low order explosion in an accident. Results show that aerosol release fraction (ARF) and respirable release fraction (RRF) of silver increase linearly with SIEI first, then varies in a range of 8% ~ 19%, with a turning point at SIEI of 1.4 MJ/kg. Analysis suggests an increase of difference between ARF and RRF with respect to SIEI. The size distribution of silver aerosol around 10 µm is influenced by SIEI significantly, which could be possibly attributed to the interactions between silver aerosol and ambient aerosol (solid HE products or rust). Comparison between the source term of silver and plutonium suggests that silver is a good surrogate of plutonium in studying aerosol for SIEI of MJ/kg level.

## Introduction

Nuclear materials are carcinogenic to humans, especially aerosolized nuclear materials can be a long-term irradiating source inside the human body. Thus, the aerosol of nuclear materials is a big concern of the public for health reasons. Aerosols from serious accidents with nuclear weapons represent a large proportion of the sources of radioactive aerosols^1–4^. To evaluate the aerosolizing amount and dispersity of radioactive materials in such accidents, a serial of field experiments called Operation Roller Coaster (ORC) were conducted in Nevada in 1963 ~ 1965^5–8^, which indicated that plutonium was completely aerosolized in these experiments^9^. However, field explosive experiments involving long-lived radioactive materials such as plutonium are now banned for political and safety reasons. As a result, a number of materials, including Ce, Ag and NaCl, have been explored as surrogates to mimic the aerosol behavior of plutonium in the safety study of nuclear materials^10–13^. Our team has studied aerosol similarity of several surrogates of plutonium including Ce, Ag and W by comparing with ORC results^14–16^. It was concluded that silver is a good surrogate for simulating the cumulative mass distribution of plutonium. Unfortunately, the experiment at that time was carried out in an explosive container, and the detonation products were extracted through a pipe and then sampled, resulting in only a normalized distribution of aerosol particles with an aerodynamic diameter (AD) of less than 10 µm being available. Therefore, comparison between the aerosol amounts of silver and plutonium is lacking, making the modeling relationship between the aerosol of silver and plutonium not fully established.

In fact, the high explosive (HE) of the warhead triggered by an accidental ignition would most probably result in a lower order explosion or namely a high explosive violent response (HEVR) in nuclear accidents^17–19^, causing a wide range of the specific internal energy input (SIEI) ranging from approximately 0.1 MJ/kg to several MJ/kg. It has been gradually discovered that SIEI affects the aerosol source term of materials in explosions, and a great deal of research has been done on the influence mechanism since the 1960s^20,21^. Sreekanth built a combined model describing both the volume expansion of a fire ball and the aerosol interactions to investigate the effect of TNT mass on the size distribution of aerosol, and the results showed that the final average particle size appears relatively insensitive to the TNT mass, due to the combined action of coagulation and volume expansion^22^. As they mainly focused on the aerosol of HE products, aerosol of metal materials was not discussed. Phalen studied the relationship between SIEI and aerosol source term using the exploding wire technique and found that the count median diameter (CMD) of aerosol particles tends to fall within a relatively narrow range, and decreases as the SIEI increases^23^. However, the SIEI involved by the exploding wire experiments normally ranged from several MJ/kg to hundreds MJ/kg, which was significantly larger than that in the lower order explosion state of the accident case (less than a few MJ/kg)^24^. Moreover, the series of plutonium material experiments conducted in the ORC mentioned above are all to simulate a full detonation scenario, and their SIEI are as high as several MJ/kg, which is the ceiling of an accident. Therefore, the design of experiments to investigate the mechanism of SIEI on the aerosol source term in an HEVR explosion is a new research topic in the field of nuclear safety.

To study the aerosol characters in the HEVR explosion, Sagartz in Sandia National Laboratory studied the silver aerosol source terms caused by different explosion intensities, using explosives with lower detonation pressure or a device with buffering layer outside the silver shell^24^. Their result indicates that there is a certain relationship between the amount of aerosolized silver and the amount of melted material. However, the SIEI in their experiments only covered the main explosion intensities in weapon cookoff accidents which are estimated to be mild or medium HEVR^19^. The influence of SIEI on the amount of silver aerosol has not been discussed as well. How the aerosol source term varies with SIEI is still empty, especially in the sever HEVR case, which could occur in shock-initiated explosions. In addition, their work did not study the size distribution of silver aerosol particles and the regularity of variation of this distribution with SIEI, which is an important characteristic when it comes to aerosol dispersion.

In this study, a serial of HE detonation devices supplying certain internal energy to the silver shell inside are designed to explore relationship between SIEI and aerosol source term through experiments. Moreover, an experimental technique for in-situ sampling of the aerosol source term inside the explosion room is established to obtain complete source data of silver aerosol under explosive loading, including the total amount of aerosol and the particle size distribution of AD below 10 µm. In addition, the direct simulation Monte Carlo (DSMC) particle simulation method is used to study the influence of coagulation and deposition effects on the aerosol source term.

## Materials and methods

### Design of HE detonation devices

The HE detonation devices were designed with numerical simulations. In the simulation model, the ignition and growth reactive burn model (IGRB) was applied to model the ignition of HE, and the Jones-Wilkins-Lee (JWL) equation of state was used to describe the detonation product of HE. The obtained values of the parameters of JWL are listed in Table 1.

**Table 1:** JWL parameters used in simulation for HE.

HE	*ρ*_0_ g/cm^3^	*D*_CJ_ km/s	*p*_CJ_ GPa	JWL parameters				
				*A* GPa	*B* GPa	*R*_1_	*R*_2_	*ω*
JH-9005	1.645	8.817	24.47	1274.038	16.322	5.44	1.82	0.22
RHT-902	1.71	7.89	27.3	602.07	12.246	4.554	1.207	0.322

**Figure 1:**
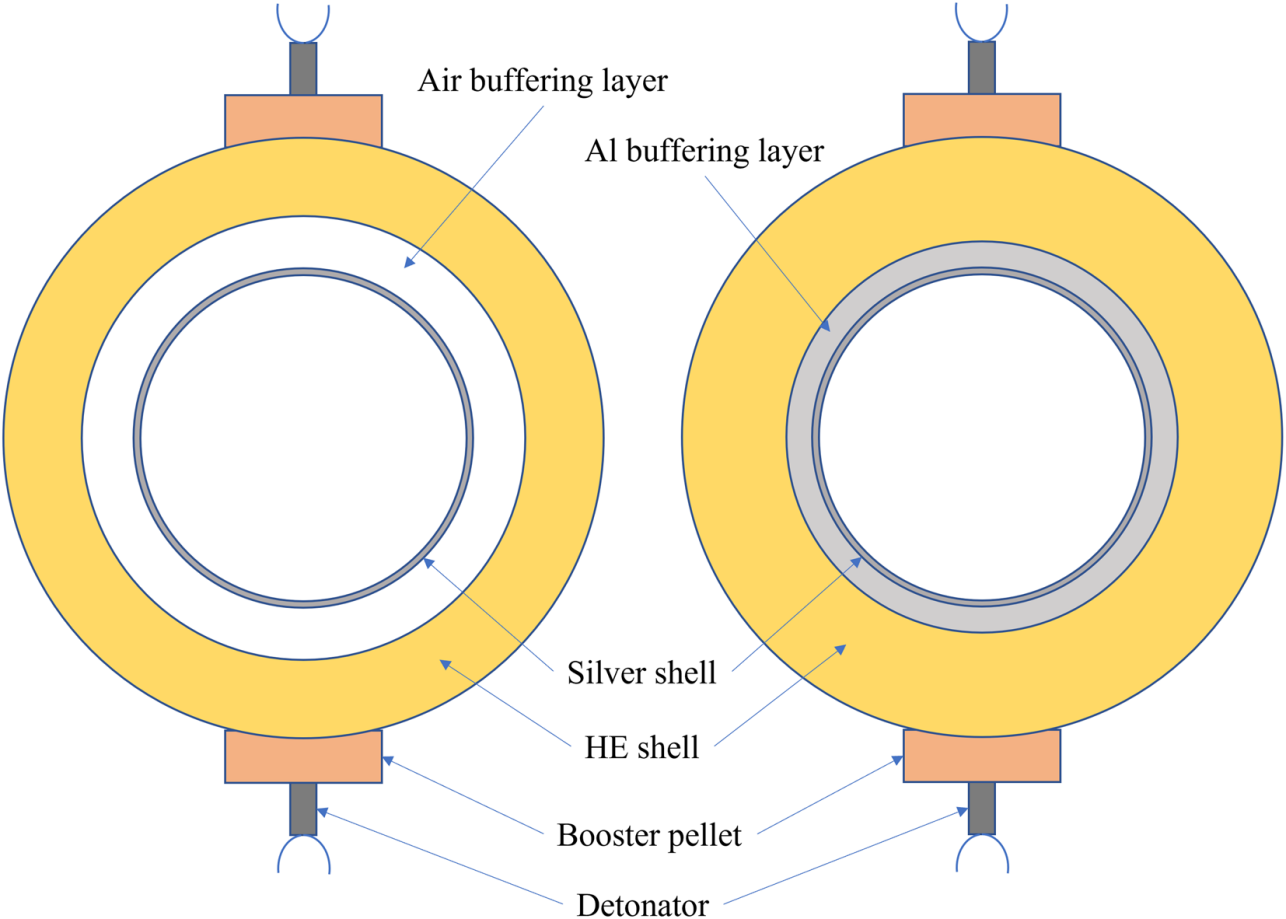
Scheme of HE detonation devices. A silver shell of 1 kg constant mass is located inside a HE (RHT902) shell. A buffering layer of air or aluminum is set between those two shells.

The basic design of the HE detonation devices are shown in Fig. 1, including buffering layer (Air or Al), silver shell, HE shell (RHT-902), booster pellets and detonators, where the buffering layer of air or aluminum was applied between silver and HE to mitigate the strength of implosion loading. The SIEI required for this experiment can be obtained by adjusting the size of the silver shell and HE shell. The values of the SIEI can range from 0.301 to 2.95 MJ/kg, and are sufficient to cover most HEVR cases. The detailed parameters of the HE detonation devices are listed in Table 2.

**Table 2:** Design of HE detonation devices.

Test number	Sizes of detonation device: sphere radius				Energy	
	Air (mm)	Silver shell (mm)	Buffering layer (mm)	HE RHT-902 (mm)	TNT equivalence (kg TNT)	Specific internal energy input (MJ/kg)
1	0 ~ 7.3	7.3 ~ 28.5	air: 28.5 ~ 95	95 ~ 115	6.0	0.301
2	0 ~ 37.16	37.16 ~ 42.0	air: 37.16 ~ 95	95 ~ 115	6.0	0.853
3	0 ~ 60.49	60.49 ~ 62.5	air: 62.5 ~ 90.8	90.8 ~ 115	7.0	1.420
4	0 ~ 60.49	60.49 ~ 62.5	air: 62.5 ~ 85	85 ~ 115	8.2	1.883
5	0 ~ 60.49	60.49 ~ 62.5	Al: 62.5 ~ 75	75 ~ 115	10.0	2.550
6	0 ~ 60.49	60.49 ~ 62.5	Al: 62.5 ~ 65	65 ~ 115	11.3	2.95

To simulate the lower order explosion in an accident, the loading strength of implosion was mitigated by applying a buffering layer of air or aluminum between silver and HE.

### Experimental details

This experiment includes six working conditions as listed in Table 2 in Sect. “Design of HE detonation devices”.

The experiment was carried out at the 40 kg TNT explosion room with an inner volume of approximately 2350 m^3^ in the Institute of Fluid Physics (IFP). Two types of samplers were used in the experiments. One is the total suspended particle (TSP) sampler, and the other is an eight-stage non-viable Andersen cascade impactor. It is generally accepted that particles with AD less than 100 µm are considered to be suspended in the air. Thus, the TSP samplers are used to collect particles with AD smaller than 100 µm, among which the Anderson cascade impactor can collect particles with AD less than 10 µm. To ensure the samplers runs correctly, necessary protections were applied to isolate the samplers from explosion shocks. Details of the above samplers are shown in the Table 3.

**Table 3:** Run parameters for the samplers used in the experiment.

Sampler type	TSP sampler	Andersen cascade impactor
Equipment name	Model TE-HIVOL + , TISCH	Model TE-20–800, TISCH
Number of samplers	4	4
AD of sampling object	< 100 µm	< 10 µm
Impactor cut points of AD (µm)	–	Stage No.0 = (9–10) Stage No.1 = (5.8–9) Stage No.2 = (4.7–5.8) Stage No.3 = (3.3–4.7) Stage No.4 = (2.1–3.3) Stage No.5 = (1.1–2.1) Stage No.6 = (0.7–1.1) Stage No.7 = (0.4–0.7) Stage No. Filter = (0–0.4)
Flow rate set	1132 L/min	28.3 L/min
Sampling time	5 min	5 min
Start time of sampling	at 30 s after explosion	at 30 s after explosion

Subsequently, the samples were collected and then analyzed by ICP-MS to derive the mass of silver. Those data were used to derive size distribution, aerosol release fraction (ARF) and respirable release fraction (RRF) of the silver aerosol. Following the instructions of Liu’s work^16^, ARF is defined as the percentage of the surrogate material that suspended in the air. RRF is defined to be the percentage of surrogate material that is respirable to human body. Generally, particle with AD smaller than 100 µm or 10 µm is considered to be suspended or respirable respectively. The ARF of silver aerosol could be given by

1 $${\text{ARF}}=\frac{{c}_{\text{Ag\_TSP}}{V}_{\text{room}}}{{m}_{\text{Ag}}}$$

where *c*_Ag_TSP_ is the silver aerosol mass concentration derived from TSP sampler, *V*_room_ represents volume of the explosion room, and *m*_Ag_ is mass of silver in the explosion device. Similarly, the RRF is given by

2 $${\text{RRF}}=\frac{{c}_{\text{Ag\_Anderson}}{V}_{\text{room}}}{{m}_{\text{Ag}}}$$

where *c*_Ag_Anderson_ is the total silver aerosol mass concentration derived from sum of silver mass on all stages of Anderson sampler.

### Coagulation simulation

After the explosion, there are mainly three components of aerosols in the explosion room, including the carbonaceous particles generated from production of HE explosion, the rust particles from steel plate and the particles originated from the materials inside the HE shell. Therefore, to derive a more comprehensive understand from the aerosol source term data varying with SIEI of silver, the DSMC method with no time counter (NTC) sampling algorithm described by Palaniswaamy^25^ was used to simulate the coagulation and deposition effect of multi-component. Three kinds of coagulation kernel (Brownian, turbulent and gravitational) were used in our simulations to account for different coagulation coefficients. This method was described in detail in their serial works, and has been proved to model multicomponent aerosols accurately^26–28^.

To verify the correctness of our DSMC program, simulation results of two test problems of deposition and coagulation processes were compared with their analytic solutions, and given in the supplementary Fig. S1 and Fig. S2.

A log-normal distribution was applied for silver particles, with CMD in AD of 0.26 µm and geometric standard deviation *σ*_g_ of 2. The volume median diameter (VMD) in AD is then CMDexp(3ln^2^*σ*_g_) = 1.1 µm, which is similar to our mass-size distribution of silver aerosol in the experiments. For the HE detonation products and rusts, a size distribution of an ultrasonic treated dust sample (collected from floor of the explosion room in a similar experiment 1 day after explosion) was adopted to mimic the initial size distribution of HE detonation products and rusts, which could be found in supplementary Fig. S3. Considering the particles larger than 100 µm contributes little to the aerosol, the size distributions of HE detonation products and rusts were limited below 100 µm. The initial settings in each simulation case were listed in Table 4. *n*_0_ is the initial number concentration of aerosols. In the DSMC, sufficient number of particles for each simulated component are needed to guarantee a meaningful statistical result, while the total number of particles is limited by computational ability. As a result, the difference between number concentrations of components would not be large. Therefore, *n*_0_ for all the three components were set to be at a common level of 10^9^ m^-3^. A scaling factor was used to limit the aerosol concentrations in simulation to a computationally acceptable level. The coagulation coefficients were multiplied by the scaling factor to give a higher concentration result in a lower concentration simulation, just as Palsmeier described in their works^28^. Particle densities of silver and rusts were estimated to be the densities of bulk silver and Fe_2_O_3_ respectively. Particle of HE detonation products was regarded as agglomeration of carbonaceous particles^29^. Its density was estimated to be nearly a half of graphite.

**Table 4:** Parameters used in DSMC simulations.

Parameters	Case 1	Case 2	Case 3	Case 4	Case 5
*n*_0_silver_ (m^-3^)	4 × 10^9^	4 × 10^9^	4 × 10^9^	4 × 10^9^	4 × 10^9^
*n*_0_HE_ (m^-3^)	8 × 10^8^	2 × 10^9^	4 × 10^9^	6 × 10^9^	8 × 10^9^
*n*_0_rust_ (m^-3^)	8 × 10^8^	2 × 10^9^	4 × 10^9^	6 × 10^9^	8 × 10^9^
Simulation time (s)	300				
Density of silver (kg/m^3^)	10.49 × 10^3^				
Density of HE products (kg/m^3^)	1.0 × 10^3^				
Density of rusts (kg/m^3^)	5.25 × 10^3^				
Scale factor	4 × 10^4^				

## Results and discussions

### Influence of SIEI on silver aerosol source term

#### Influence of SIEI on size distribution of silver aerosol

**Figure 2:**
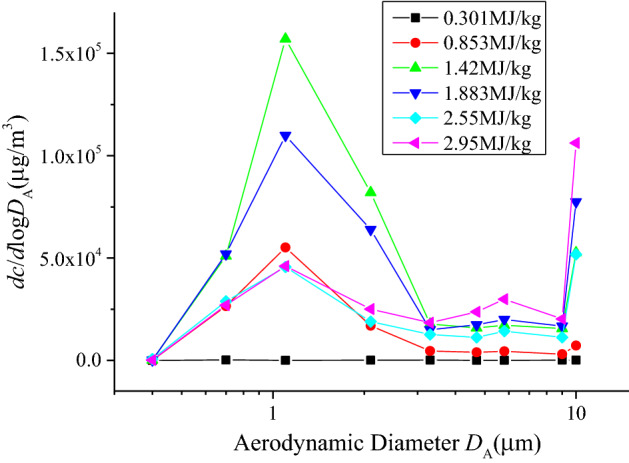
Mass concentration-size distribution of silver aerosol at different specific internal energy input. The concentrations were calculated by assuming the spatial distribution of aerosols was uniform, since strong turbulences were produced in the explosion room right after the explosion.

**Figure 3:**
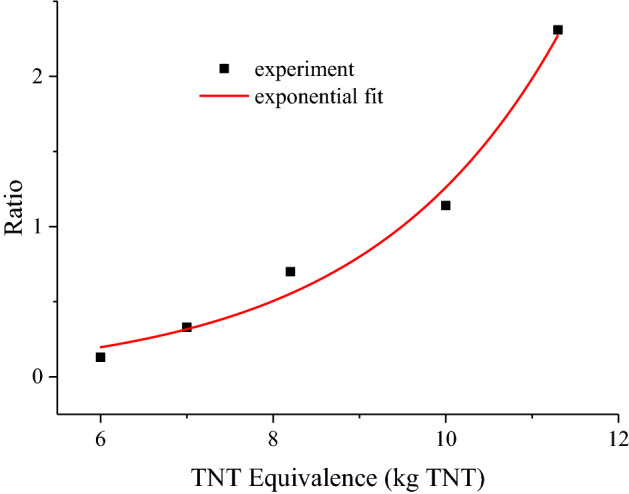
Ratio of mass concentration-size distribution of silver aerosol between 10 and 1.1 µm, versus TNT equivalence. As the sampled mass of silver aerosol for 0.301 MJ/kg is too low to detect accurately, the data for 0.301 MJ/kg is not given. An exponential fit is applied and presented by red line, which shows a clear exponential increase tendency of the data points.

To explore the influence of SIEI on size distribution of silver aerosol, mass concentration-size distributions for test 1 ~ 6 are calculated and drawn in Fig. 2. The mass concentration-size distribution is given by d*c*_Ag_/dlogAD, according to the AD intervals in each sampling stage, where *c*_Ag_ is the mass concentration of silver aerosol calculated by *m*_s_/*V*_s_. *m*_s_ is the mass of silver aerosol collected in a certain AD range, and is the average of four Anderson samplers (The original mass of silver results for each sample could be found in the supplementary Table S1). *V*_s_ is sampling volume. According to Fig. 2, all tests show clear peaks in the mass concentration-size distribution at around 1.1 µm, with the exception of 0.301 MJ/kg, where the silver aerosol is too low to be accurately detected, indicating the same pattern of production of these particles. Moreover, there is a significant distribution at 10 µm, which hints another mode of silver aerosol production that distinguishes from the 1.1 µm mode. Ratio between mass concentration-size distribution at 10 µm and 1.1 µm is calculated and drawn in Fig. 3 with respect to the TNT equivalence, where the data of 0.301 MJ/kg is absent for the reason of low mass concentration of silver aerosol. In Fig. 3, an exponential relation between the ratio and the TNT equivalence is indicated. Those results show that the production of larger particles of silver aerosol grows as the TNT equivalence increases. The reason could be that as the HE of device increases, total amount of aerosol increases, especially those carbonaceous particles is always large and porous that produced by the HE explosion^29^. Those aerosol particles increase the possibility of coagulation of silver aerosol, consequently resulting in a higher distribution of larger silver aerosol particles.

In the above, it is suspected that the way in which TNT equivalence affects the size distribution of silver aerosols is by coagulation. To test this hypothesis and gain a clearer picture of trends, the following simulation and analyses of the coagulation and deposition effects of multicomponent aerosols are performed.

**Figure 4:**
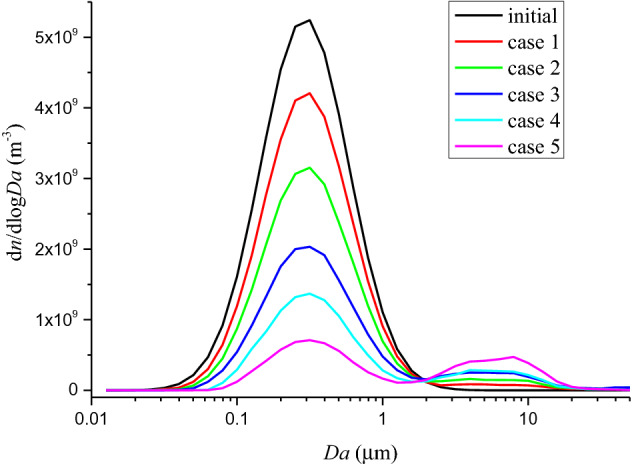
Number concentration-size distribution of silver aerosol for DSMC simulation case 1 to case 5 at 300 s.

**Figure 5:**
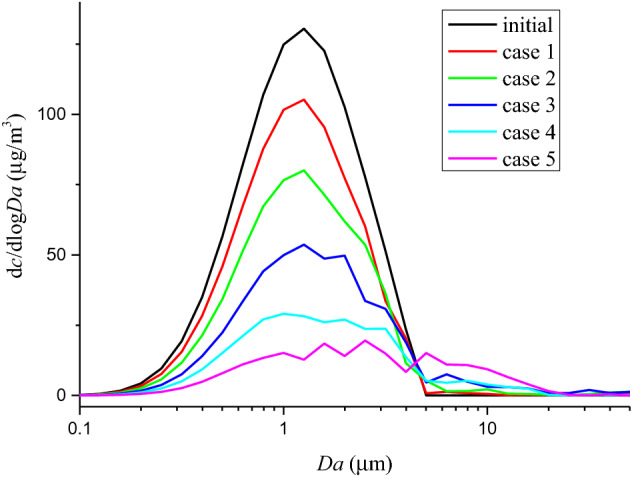
Mass concentration-size distribution of silver aerosol for DSMC simulation case 1 to case 5 at 300 s.

**Figure 6:**
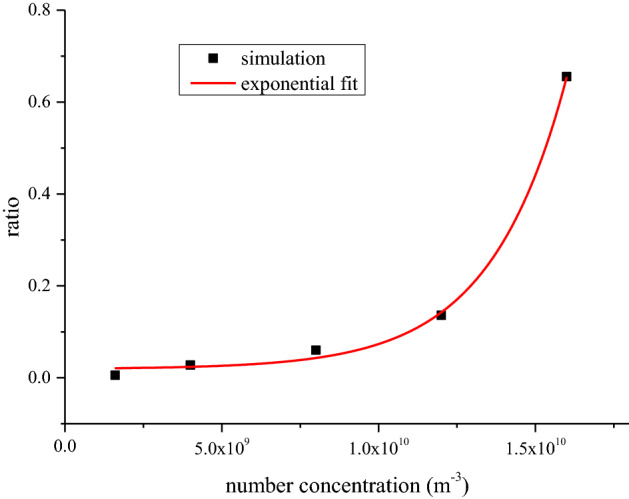
Ratio of mass concentration-size distribution of silver aerosol between 10 and 1.1 µm, versus the total number concentration of HE products and rust aerosols. An exponential fit is applied and presented by red line, which shows a clear exponential increase tendency of the data points.

The DSMC simulation results are presented in Figs. 4 and 5 for mass concentration-size distribution and number concentration-size distribution of silver aerosol respectively. In Figs. 4 and 5, a clear peak could be seen around the larger particle zone (~ 10 µm) for both number concentration-size distribution and mass concentration-size distribution. By increasing the HE detonation product particle and rust particle, the amount of size distribution at 10 µm increased dramatically as the experimental results did. The ratios between mass concentration-size distributions at 1.1 µm and 10 µm for simulation case 1 to case 5 were calculated and drawn in Fig. 6. The simulated results show an exponential increasing of the ratio with increasing the total number concentration of HE products and rust aerosols. Considering a linear increase of aerosol of surrounding materials was expected with increasing the HE amount^30^, the above simulation results agree well with the experimental tendency in Fig. 3. The similar tendencies of the simulation and the experiment suggest the mechanism behind those experimental observations is possibly originated from coagulation effect. Therefore, it could be deduced that SIEI may influence the size distribution of silver aerosol via the varying amount of HE, which changes the aerosol environment, where the coagulation between silver aerosol particles and ambient aerosol particles changes the size distribution of silver aerosol significantly.

#### Influence of SIEI on ARF and RRF of silver aerosol

**Figure 7:**
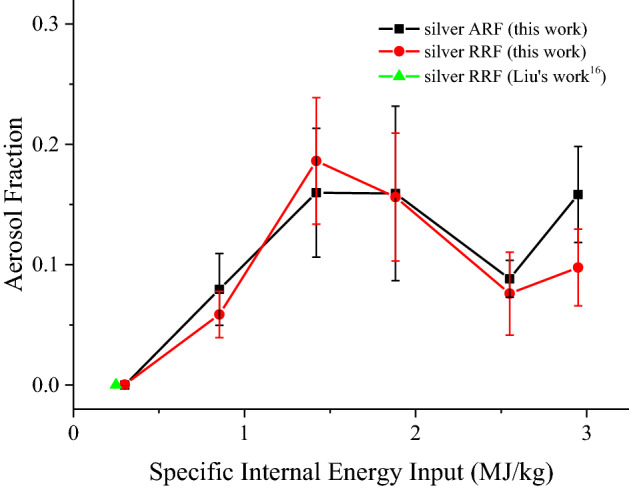
Aerosol release fraction and respirable release fraction of silver aerosol versus specific internal energy input. Error bars were derived from the root mean square of mass concentrations in four TSP or Anderson samplers, when calculating the averages of those concentrations. The RRF of silver aerosol in the former work^16^ is also included for comparison.

The ARF and RRF of silver aerosol are calculated from average mass concentrations of silver of TSP and Anderson samplers respectively. (The original mass of silver as well as the sampling volume for each TSP sample could be found in the supplementary Table S2.) The results are shown in Fig. 7 and compare these with the previous experimental results by others. Error bars are derived from the root mean square of mass concentrations in 4 TSP or Anderson samplers when these concentrations are averaged. The fluctuations of these results would be caused by the non-ideal sampling conditions such as the nonuniform distribution of aerosol in the explosion room, changes in sampling flow rate and silver measurement errors.

As shown in Fig. 7, the ARF and RRF of silver increased linearly with SIEI less than 1.4 MJ/kg. The RRF result point from the previous experiment is consist with this linear increasing trend as well. In the range of SIEI larger than 1.4 MJ/kg, the linear increasing tendencies of ARF and RRF stop, a variation band in the range of 8% ~ 19% is observed. Due to the application of aluminum buffering layers, both ARF and RRF fluctuated at SIEI of 2.55 MJ/kg and 2.95 MJ/kg, respectively. These fluctuations may be caused by the asymmetry implosion load from aluminum buffering layer at certain thickness. According to the definition of ARF and RRF described in Sect. “Experimental details”, difference between ARF and RRF indicates the mass fraction of aerosol in the AD range of 10 µm ~ 100 µm. It is observed in Sect. “Influence of SIEI on size distribution of silver aerosol” that the distribution of larger silver particles (AD ~ 10 µm) increases exponentially with SIEI, leading to a quick increase of the difference between ARF and RRF of silver aerosol with the increase of SIEI. From Fig. 7, compared with other data points, only the difference between ARF and RRF at the largest SIEI of 2.95 MJ/kg is equivalent with the error value, which is a support for the deduction in Sect. “Influence of SIEI on size distribution of silver aerosol”.

In general, the changes of ARF and RRF with the increase of SIEI are mainly divided into two stages. When SIEI is less than 1.4 MJ/kg, both ARF and RRF increase linearly with the increase of SIEI. With the increase of SIEI larger than 1.4 MJ/kg, ARF and RRF stop the increasing tendency with respect to SIEI, and varying in the range of 8% ~ 19%. Moreover, an increase of the difference between ARF and RRF with SIEI is suggested by the results in Fig. 7.

### Comparison between ARF and RRF of silver and plutonium

In the former work we have concluded that silver could be used as a surrogate of plutonium to simulate the cumulative mass fraction of plutonium aerosol in HE explosions, by comparing the cumulative mass fractions of silver and plutonium aerosols^16^. To further explore the feasibility of silver to simulate the amount of plutonium aerosol in HE explosions, the ARF and RRF of silver and plutonium are compared.

**Figure 8:**
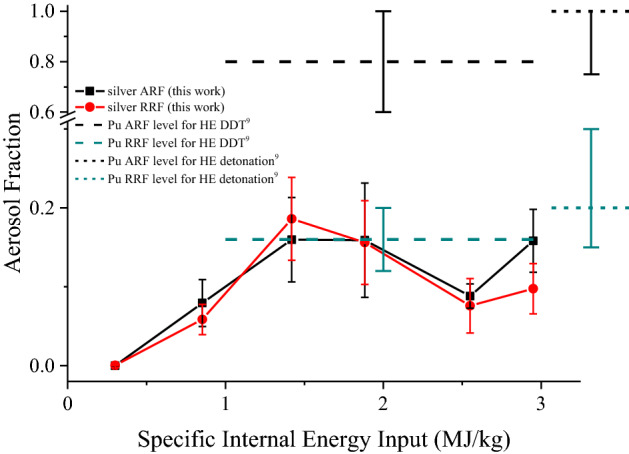
Aerosol release fraction and respirable release fraction of silver aerosol versus specific internal energy input. The ARF and RRF of plutonium with their upper and lower limits for both full and lower order detonation conditions in Stephens’ work^9^ (indicated by dashed or short dashed horizontal lines) are included for comparison.

The ARF and RRF values of plutonium are from Stephens’ work. They summarized several studies on the aerosol of plutonium in explosion, and gave the expectation value of ARF and RRF of plutonium with their upper and lower limits for both the cases of HE detonation and deflagration detonation transition (DDT). Among those results, the results for the case of HE detonation were mainly extracted from the ORC experiments^5^, a SIEI exceeding 3 MJ/kg is expected for this case. The results for the case of DDT were mainly extrapolations of the results of detonation case. The SIEI for this case is estimated at the level of (1 MJ/kg ~ 3 MJ/kg). For the clearness of the comparison between ARF and RRF of silver and plutonium, the results of plutonium from Stephens’ work together with the ARF and RRF results of silver in this work are shown in Fig. 8. From Fig. 8, the ARF of plutonium could be as high as 80% ~ 100% in both the detonation and DDT case (SIEI > 1 MJ/kg), which is much higher than the ARF of silver (8% ~ 19%) in the same SIEI range. The large gap hinders the flexibility to simulate the ARF of plutonium by ARF of silver in the case of detonation and DDT. To compare the RRF of silver and plutonium in the case of detonation (SIEI > 3 MJ/kg), although there is no data of silver in this SIEI range, an estimation could be made for the RRF of silver, from the existing data: In the case of detonation, more SIEI are available to the melting or vaporizing of silver, comparing with that in the case of DDT. According to the results that the amount of aerosol is related to the amount of melted material in Sagartz’s work^24^, and the tendency for the RRF of silver in the SIEI range of 0.3 MJ/kg ~ 3 MJ/kg, it could be asserted that the RRF of silver in detonation case would not less than the RRF of silver at elevated SIEI (1.4 MJ/kg ~ 3 MJ/kg), which varies between 8% and 19%. Therefore, a lower limit of 8% for the RRF of silver in the case of detonation is established. Meanwhile, according to the results that the aerosolization of metals are significantly influenced by the thermal and chemical properties of metal in Harper’s work^31^, the plutonium with lower melting point, lower heat of fusion, higher reactivity would result in a higher amount of aerosol, comparing with silver. Therefore, the RRF of plutonium (20%) in the case of detonation could be adopted as the upper limit for RRF of silver in the same case. By comparing those estimated lower and upper limits of silver (8% and 20% respectively) to the RRF level of plutonium (20%), it could be concluded that the RRF of silver is of the same order of magnitude with the RRF of plutonium in the case of detonation. In the case of DDT where SIEI is approximately 1 MJ/kg ~ 3 MJ/kg, RRF of plutonium estimated by Stephens is in the range of 12% ~ 20%, with an expectation value of 16%. In the same SIEI range, the RRF of silver varies between 6% and 19%. The RRF values of silver and plutonium are of the same order of magnitude as well in the case of DDT.

In the background of nuclear accident response, the magnitude of the radioactive aerosol is regarded as an important reference data to help decision making. Therefore, in the context of nuclear accident response, silver is recommended to be the surrogate of plutonium to simulate RRF of plutonium at SIEI of MJ/kg level, according to the fact that RRF values of silver and plutonium are of the similar magnitude in the case of HE detonation and DDT.

## Conclusions

A series of HE detonation devices were reasonably designed under the guidance of numerical simulations, and data on the total silver aerosol mass concentration and mass concentration-size distribution of silver with AD below 10 µm are obtained through the in-situ sampling technique in the explosion room. The experimental results are compared and analyzed with the numerical simulation results to explore the relationship between SIEI and the aerosol source term. The following conclusions can be obtained.

1. Experimental results show that ARF and RRF of silver increase with respect of SIEI linearly when SIEI is less than 1.4 MJ/kg; while with the increase of SIEI ranging from 1.4 to 3 MJ/kg, the ARF and RRF of silver stop the linear increasing, and vary in a certain range (8% ~ 19%). An increasing tendency of difference between ARF and RRF of silver with increasing SIEI is further hinted by analysis of the results. Meanwhile, SIEI influences mass concentration-size distribution of silver aerosol at 10 µm significantly. A further DSMC simulation suggests that the influence of SIEI on size distribution of silver aerosol would be most probably due to the interactions between silver aerosol and ambient aerosols which could be the solid particles of HE products and the rust particles involved in the explosion.
2. RRF of silver is recommended to simulate that of plutonium at SIEI of MJ/kg level (mainly corresponding to the HE detonation case and DDT), as analysis indicates a similarity between magnitudes of those two at such SIEI values. Additionally, considering the conclusion in the former work that size distribution of silver could simulate that of plutonium in HE explosions, it could be further concluded that silver would be a good surrogate of plutonium at such SIEI of MJ/kg level (detonation or DDT case) to study the source term and dispersal patterns of plutonium after a nuclear accident, which can provide important reference data for decision-making in the event of a radiological accident emergency.

## Supplementary Information

Supplementary Information.

## Data Availability

The data that support the findings of this study are supplied in the article and the supplementary, which is available for people.
