# Supplementary material for: Study of silver aerosol source term at different specific internal energy input from HE detonation devices
Source: Sci Rep. 2022 Feb 24;12:3148. doi: 10.1038/s41598-022-07180-w (PMC8873271; doi:10.1038/s41598-022-07180-w)
Supplement: Supplementary file 1 — Supplementary Information. [file 41598_2022_7180_MOESM1_ESM.docx]

Study of Silver Aerosol Source Term at Different specific internal energy input from HE Detonation Devices

Song Kefeng^1^, Shi Yaqin^1^*, Liu Kun^1^, Su Luochuan^1^, Li Bo^1^, Liu Wei^1^, Wang Penglai^1^, Yi Chenhong^1^, Zhang Yajun^1^, Ma Qingpeng^1^, Hu Haibo^2^, Liu Wenjie^1^*

**Table S1.** Mass of silver on Anderson sampler^a^ (unit: μg)

| Test No. | Sampler No. | Stage 0 | Stage 1 | Stage 2 | Stage 3 | Stage 4 | Stage 5 | Stage 6 | Stage 7 | Stage F |
| --- | --- | --- | --- | --- | --- | --- | --- | --- | --- | --- |
| 1 | A1 | 1.1 | 6.7 | 1.0 | 1.0 | 1.6 | 5.7 | 2.4 | 7.9 | 2.0 |
|  | A2 | 1.4 | 2.9 | 0.5 | 1.4 | 15.5 | 3.1 | 1.3 | 1.6 | 0.4 |
|  | A3 | 0.9 | 0.8 | 0.8 | 1.2 | 2.2 | 4.2 | 2.1 | 22.1 | 0.5 |
|  | A4 | 0.4 | 0.4 | 0.3 | 2.6 | 0.5 | 1.2 | 0.6 | 0.8 | 0.7 |
| 2 | A1 | 23.5 | 38.8 | 31.7 | 63.8 | 122.8 | 552.8 | 978.5 | 666.4 | 6.6 |
|  | A2 | 81.9 | 133.4 | 108.7 | 177.0 | 253.3 | 1206.2 | 2198.6 | 736.9 | 11.3 |
|  | A3 | 22.2 | 71.8 | 15.6 | 28.3 | 44.5 | 315.6 | 1021.1 | 1134.4 | 19.3 |
|  | A4 | 59.6 | 86.0 | 72.5 | 76.6 | 91.7 | 619.9 | 1930.8 | 1108.0 | 24.3 |
| 3 | A1 | 340.2 | 476.4 | 302.2 | 384.6 | 631.6 | 5088.9 | 5888.4 | 1308.7 | 19.9 |
|  | A2 | 389.9 | 421.6 | 260.5 | 452.9 | 670.9 | 4596.0 | 4923.6 | 1246.5 | 24.9 |
|  | A3 | 294.0 | 297.4 | 123.9 | 219.4 | 313.3 | 1436.9 | 2521.3 | 2021.5 | 41.6 |
|  | A4 | 338.0 | 488.2 | 200.6 | 320.7 | 354.4 | 1916.3 | 4113.7 | 2442.0 | 52.3 |
| 4 | A1 | 509.7 | 579.1 | 269.2 | 363.7 | 316.9 | 3485.8 | 4122.4 | 1799.0 | 20.9 |
|  | A2 | 553.0 | 400.9 | 316.9 | 456.9 | 672.3 | 4437.7 | 4258.9 | 1355.8 | 15.6 |
|  | A3 | 384.4 | 389.7 | 198.1 | 246.6 | 273.8 | 537.2 | 1245.1 | 2105.2 | 49.3 |
|  | A4 | 559.3 | 435.2 | 249.4 | 441.8 | 402.3 | 1699.2 | 2587.1 | 1890.4 | 46.7 |
| 5 | A1 | 241.2 | 225.1 | 129.4 | 187.1 | 225.8 | 416.9 | 569.2 | 466.7 | 71.4 |
|  | A2 | 285.9 | 229.6 | 178.7 | 195.4 | 298.3 | 429.6 | 753.3 | 631.4 | 109.2 |
|  | A3 | 371.8 | 334.7 | 166.6 | 317.5 | 356.7 | 892.6 | 1878.4 | 1399.2 | 147.9 |
|  | A4 | 443.0 | 429.4 | 263.8 | 274.8 | 526.6 | 1274.5 | 1867.3 | 1472.8 | 267.5 |
| 6 | A1 | 729.3 | 765.6 | 464.0 | 713.2 | 647.4 | 1152.0 | 2370.3 | 955.1 | 48.4 |
|  | A2 | 857.9 | 766.1 | 583.7 | 627.9 | 637.4 | 1301.9 | 1215.8 | 937.8 | 31.5 |
|  | A3 | 407.9 | 171.7 | 170.7 | 220.6 | 265.7 | 438.6 | 802.3 | 1019.2 | 25.3 |
|  | A4 | 755.3 | 485.7 | 325.4 | 500.3 | 495.2 | 1087.4 | 728.3 | 794.4 | 55.9 |

a: The sampling volumes for all the Anderson sampler were 141.5 L.

**Table S2.** Sampling result for TSP sampler

| Test No. | Sampler No. | Mass of silver  μg | Sampling volume  m^3^ | Test No. | Sampler No. | Mass of silver  μg | Sampling volume  m^3^ |
| --- | --- | --- | --- | --- | --- | --- | --- |
| 1 | T1 | 11.8 | 5.094 | 4 | T1 | 397265 | 3.594 |
|  | T2 | 21.0 | 4.641 |  | T2 | 212531 | 5.32 |
|  | T3 | 13.0 | 5.122 |  | T3 | 278242 | 5.377 |
|  | T4 | 9.0 | 4.613 |  | T4 | 375345 | 5.434 |
| 2 | T1 | 127126 | 5.037 | 5 | T1 | 248945 | 5.434 |
|  | T2 | 114387 | 5.122 |  | T2 | 175891 | 5.49 |
|  | T3 | 140013 | 3.707 |  | T3 | 187169 | 5.745 |
|  | T4 | 214281 | 5.122 |  | T4 | 224622 | 5.603 |
| 3 | T1 | 239708 | 4.924 | 6 | T1 | 446458 | 4.896 |
|  | T2 | 209444 | 2.434 |  | T2 | 326424 | 5.349 |
|  | T3 | 218835 | 2.462 |  | T3 | 341268 | 5.151 |
|  | T4 | 240434 | 4.981 |  | T4 | 275089 | 5.349 |

**Figure S1.** Validation of DSMC procedure using the deposition effect of log-average distributed rust particles. The number of particle after a time of *t* could be expressed as *N*=*N*_0_exp(-*v*_g_*t*/*H*), where *v*_g_ is the particle settling velocity: *v*_g_=*ρ*_p_*d*_p_^2^*gC*_c_/(18*η*).

**Figure S2.** Validation of DSMC procedure using the coagulation problem of mono- dispersed particles with a constant coagulation coefficient *β*. The analytical solution to this problem was given by Lushnikov (Lushnikov, 1973):

*N*(*g*, *t*)/ *N*_0_= *t’^g^*^-1^/( *t’*+1)*^g^*^+1^, where *g* is the size of the coagulated particle which means the present particle consists of *g* monomers, *t’* is a dimension less parameter: *t’* = 0.5*N*_0_*βt*.

Lushnikov, A. A. Evolution of coagulating systems. J. Colloid Interf. Sci. **45,** 549-556 (1973)

**Figure S3.** The number-size distribution of ultrasonic dispersed dust sample collected from the floor of explosion room in a similar experiment 1 day after explosion. The distribution below aerodynamic diameter of 100 μm was adopted for HE detonation products and rust aerosol distribution in the DSMC simulations.
